# Modeling Individual Differences in Children’s Information Integration During Pragmatic Word Learning

**DOI:** 10.1162/opmi_a_00069

**Published:** 2022-12-16

**Authors:** Manuel Bohn, Louisa S. Schmidt, Cornelia Schulze, Michael C. Frank, Michael Henry Tessler

**Affiliations:** Department of Comparative Cultural Psychology, Max Planck Institute for Evolutionary Anthropology, Leipzig, Germany; Leipzig Research Center for Early Child Development, Leipzig University, Leipzig, Germany; Department of Educational Psychology, Faculty of Education, Leipzig University, Leipzig, Germany; Department of Psychology, Stanford University, Stanford, USA; DeepMind, London, UK; Department of Brain and Cognitive Sciences, Massachusetts Institute of Technology, Cambridge, USA

**Keywords:** pragmatics, language development, individual differences, cognitive modeling

## Abstract

Pragmatics is foundational to language use and learning. Computational cognitive models have been successfully used to predict pragmatic phenomena in adults and children – on an aggregate level. It is unclear if they can be used to predict behavior on an individual level. We address this question in children (*N* = 60, 3- to 5-year-olds), taking advantage of recent work on pragmatic cue integration. In Part 1, we use data from four independent tasks to estimate child-specific sensitivity parameters to three information sources: semantic knowledge, expectations about speaker informativeness, and sensitivity to common ground. In Part 2, we use these parameters to generate participant-specific trial-by-trial predictions for a new task that jointly manipulated all three information sources. The model accurately predicted children’s behavior in the majority of trials. This work advances a substantive theory of individual differences in which the primary locus of developmental variation is sensitivity to individual information sources.

## INTRODUCTION

A defining feature of human communication is its flexibility. Conventional languages – signed and spoken – allow for expressing a near-infinite number of messages. In the absence of a shared language, humans can produce and understand novel signals which can rapidly be transformed into structured communication systems (Bohn et al., 2019; Brentari & Goldin-Meadow, 2017; Fay et al., 2018). The flexibility stems from a powerful social-cognitive infrastructure that underlies human communication (Levinson & Holler, 2014; Sperber & Wilson, 2001; Tomasello, 2008). Interlocutors can recruit and integrate a range of different information sources – conventional language being one of them – to make so-called *pragmatic* inferences about the speaker’s intended meaning in context (Grice, 1991). They play an important role during everyday language use (Clark, 1996; Schulze & Buttelmann, 2021) and during language acquisition (Bohn & Frank, 2019; Clark, 2009; Tomasello, 2009).

Decades of developmental research have shown that children readily make pragmatic inferences in a wide variety of contexts and starting at an early age (Bohn & Frank, 2019; Schulze & Tomasello, 2015). For example, already early in the second year of life, children use their emerging semantic knowledge (word-object mappings) to infer that a speaker uses a novel word to refer to a novel object (Bion et al., 2013; Clark, 1988; Halberda, 2003; Lewis et al., 2020; Markman & Wachtel, 1988; Merriman et al., 1989; Pomiechowska et al., 2021). Around the same age, children start to use common ground (shared knowledge) in communication (Akhtar et al., 1996; Bohn & Köymen, 2018; Bohn et al., 2018; Diesendruck et al., 2004; Ganea & Saylor, 2007). From age three onwards, they expect speakers to communicate in an informative and context-sensitive way (Frank & Goodman, 2014; Schulze et al., 2022; Schulze et al., 2013).

Theoretical accounts of language use and learning postulate that these pragmatic inferences require integrating various sources of information but often fail to specify how exactly the information integration happens. This theoretical paucity is a special case of a more general issue in psychology and – specifically — in developmental science, where there is a lack of strong, explicit theories that predict and explain behavior (Muthukrishna & Henrich, 2019). Computational cognitive modeling is one way to overcome this issue (van Rooij & Baggio, 2021; Simmering et al., 2010). Cognitive models formalize the computational processes that generate the observed behavior (Ullman & Tenenbaum, 2020; van Rooij, 2022). The modeling process forces researchers to state explicitly their assumptions and intuitions, which can result in stronger theories (Guest & Martin, 2021).

The field of pragmatic language comprehension has been particularly active from a computational modeling perspective (Cummins & de Ruiter, 2014), including work on common ground (Anderson, 2021; Heller et al., 2016), politeness (Yoon et al., 2020); over-informativeness (Degen et al., 2020); implicature (Franke & Bergen, 2020), and generic language (Tessler & Goodman, 2019). The Rational Speech Act (RSA) framework has been one productive framework for modeling pragmatic inference, construing language understanding as a special case of Bayesian social reasoning (Frank & Goodman, 2012; Goodman & Frank, 2016; Scontras et al., 2021). RSA models are distinguished by their recursive structure in which a listener reasons about a cooperative speaker – sensu Grice (1991) – who reasons about a literal listener who interprets words according to their literal semantics. These models have been successfully applied to predict aggregate behavior – the average judgment probability across a large group of participants, for example – for a range of different pragmatic phenomena (reviewed in Frank & Goodman, 2012; Goodman & Frank, 2016).

Computational cognitive models – including RSA – are mostly used as summary descriptions and explanations of well-known effects from the literature or in pre-existing data. Yet, for a comprehensive theory, models should also be able to *predict* new data (Hofman et al., 2021; Shmueli, 2010; Yarkoni & Westfall, 2017). Recent work using RSA models has begun to address this issue. For example, Bohn et al. (2021) studied young children’s information integration during pragmatic word learning (see also Bohn et al., 2022b). They measured children’s developing sensitivity to three different sources of information about meaning in context and used an RSA model to generate predictions about situations in which these information sources need to be integrated. Newly collected data aligned closely with what the model predicted, in the sense that the model predictions were numerically similar to the average level of performance across a large sample of children. This line of work tested the scope and validity of models of pragmatic reasoning and the results offered support for the theoretical assumptions around which the model was built in comparison to alternative models.

These prior studies only explained and predicted behavior on an *aggregate* level, however. The models were assessed following the assumption that the “average person” behaves like the prototypical agent whose cognitive processes are being simulated by the model (Estes & Todd Maddox, 2005). Yet it is an open question if everybody – or in fact anybody – really behaves like this prototypical agent. Most likely, there are differences between individuals. For example, Franke and Degen (2016) studied quantity implicatures and found that participant data was best captured by a model that assumes a population in which individuals differ in the depth of their Theory of Mind reasoning. A central question is, therefore, whether models that accurately predict group-level results can also be used to predict individual differences. For example, although Griffiths and Tenenbaum (2006) showed that groups of participants in the aggregate could correctly make optimal judgments about the conditional probability of everyday events, Mozer et al. (2008) argued that this pattern could emerge from an aggregate of individual agents with far simpler and more heuristic strategies (cf. Griffiths & Tenenbaum, 2011). Thus, the fit of cognitive models to aggregate patterns of data may not always support the inference that the cognitive model describes individuals’ patterns of reasoning or inference.

In the present study, we address this issue in the domain of pragmatic word learning, using RSA models to predict individual differences between children. Our study builds on Bohn et al. (2021) and measures how children integrate different information sources. We focused on how children’s semantic knowledge interacts with their expectations about informative communication and sensitivity to common ground. Following the previous study, we formalized this integration process in a model derived from the RSA framework. Importantly, however, the current model was designed to capture individual differences, which we conceptualize as differences between children in sensitivity to the different information sources. In Part 1, we collected data in four tasks from which we estimated child-specific sensitivity parameters. In Part 2, we used these parameters to predict – on a trial-by-trial basis – how the same children should behave in a new task that required information integration. The critical contribution of this work is thus to test whether a successful model of aggregate judgments holds at the individual level.

## PART 1: SENSITIVITY

### Methods

Methods, sample size, and analyses were pre-registered at: https://osf.io/pa5x2. All data, analysis scripts, model code, and experimental procedures are publicly available in the following online repository: https://github.com/manuelbohn/spin-within.

#### Participants.

We collected complete data for 60 children (*m*_*age*_ = 4.11, range_*age*_: 3.06–4.93, 30 girls) during two experimental sessions each. As per our pre-registration, children who provided valid data for fewer than half of the test trials in any of the three experiments were excluded from the analysis. This was the case for five additional children (two 3-year-olds, three 4-year-olds) due to disinterest in the experiments (*n* = 2), parental interference due to fussiness (*n* = 2), or withdrawal from the study after the first testing session (*n* = 1). Children came from an ethnically homogeneous, mid-size German city (∼550,000 inhabitants, median income €1,974 per month as of 2020), were mostly monolingual, and had mixed socioeconomic backgrounds. The study was approved by an internal ethics committee at the Max Planck Institute for Evolutionary Anthropology. Data was collected between March and July of 2021.

#### Measures.

**Figure 1.**
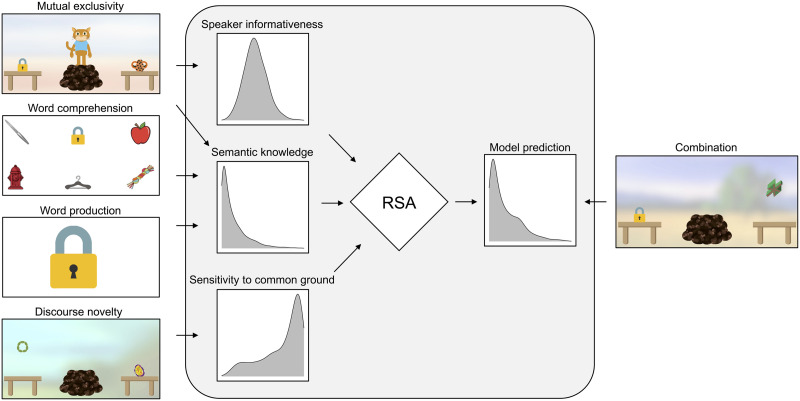
**Schematic overview of the study and the model.** Pictures on the left show screenshots from the four sensitivity tasks. Arrows indicate which tasks informed which parameter in the model (grey area). Based on the data from the sensitivity tasks, child-specific parameter distributions for each information source were estimated. These sources were integrated via an RSA model, which generated predictions for each trial of the combination task. These predictions were then evaluated against new data from the combination task.

Children were recruited via a database and participated with their parents via an online conferencing tool. The different tasks were programmed as interactive picture books in JavaScript/HTML and presented on a website. During the video call, participants would enter the website with the different tasks and share their screens. The experimenter guided them through the procedure and told caregivers when to advance to the next task. Children responded by pointing to objects on the screen, which their caregivers would then select for them via mouse click. For the word production task, the experimenter shared their screen and presented pictures in a slide show. For the mutual exclusivity, discourse novelty, and combination tasks (Part 2), pre-recorded sound files were used to address the child. Figure 1 shows screenshots from the different tasks.

The *discourse novelty* task assessed children’s sensitivity to common ground (see Figure 1). Children saw a speaker (cartoon animal) standing between two tables. On one table, there was a novel object (drawn for the purpose of this study), while the other was empty (side counterbalanced). The speaker sequentially turned to both sides (order counterbalanced) and either commented on the presence or absence of an object (without using any labels, see supplementary material for details). Then, the speaker disappeared, and – while the speaker was gone – another novel object appeared on the previously empty table. Next, the speaker re-appeared and requested one of the objects using a novel non-word as the label. We assumed that children would take the novel word to refer to the object that was new to the speaker. Children received 12 trials, each with a new pair of novel objects.

The *mutual exclusivity* task was used to assess children’s semantic knowledge and expectations about speaker informativeness (see Figure 1). Children again saw a speaker and two tables. On one table, there was a novel object while on the other there was a (potentially) familiar object (side counterbalanced). The speaker used a novel non-word to request one of the objects. We assumed that children would take the novel word to refer to the novel object. In line with previous work (Bohn et al., 2021; Grassmann et al., 2015; Lewis et al., 2020) we assumed this inference would be modulated by children’s lexical knowledge of the familiar object. Children received 16 trials, each with a new pair of novel and familiar objects. Both the discourse novelty as well as the mutual exclusivity tasks showed good re-test reliability (*r* > .7 for both tasks) in a previous study and seem well-suited for individual-level measurement (Bohn et al., 2022a).

The *word production* task assessed children’s semantic knowledge (see Figure 1). The experimenter showed the child each of the 16 familiar objects from the mutual exclusivity task and asked them to name them. We used a pre-defined list of acceptable labels per object to categorize children’s responses as either correct or incorrect (see supplementary material).

The *word comprehension* task was also used to assess semantic knowledge (see Figure 1). The child saw four slides with six objects. Four objects per slide were taken from the 16 familiar objects that also featured in the mutual exclusivity and word production tasks. Two objects were unrelated distractors. The experimenter labeled one familiar object after the other and asked the child to point to it.

Data collection for the entire study (Part 1 and 2) was split into two sessions which took place around one week apart (min: 1 day, max: 2 weeks). On day one, children completed the mutual exclusivity and the discourse novelty tasks. On day two, they completed the combination task (Part 2) followed by the word comprehension and production tasks.

### Analysis

The goal of the analysis of Part 1 was to estimate participant-specific sensitivity parameters based on the tasks described above. Parameter estimation happens in the context of the modeling framework we used to generate predictions for the novel task in Part 2. In the following, we first describe the general modeling framework and then continue with the participant-specific parameter estimation.

#### Modeling Framework.

We adopted the modeling framework used by Bohn et al. (2021). Our models are situated in the Rational Speech Act (RSA) framework (Frank & Goodman, 2012; Goodman & Frank, 2016). RSA models treat language understanding as a special case of Bayesian social reasoning. A listener interprets an utterance by assuming it was produced by a cooperative speaker who has the goal to be informative. Being informative is defined as producing messages that increase the probability of the listener inferring the speaker’s intended message. The focal *rational integration* model, including all data-analytic parameters, is formally defined as:

$$P_{L_{1}}\left(r|u;\left\{\rho_{i},\alpha_{i},\theta_{ij}\right\}\right)\propto P_{S_{1}}\left(u|r;\left\{\alpha_{i},\theta_{ij}\right\}\right)\cdot P\left(r|\rho_{i}\right)$$ (1)

The model describes a listener (*L*_1_) reasoning about the intended referent of a speaker’s (*S*_1_) utterance. This reasoning is contextualized by the prior probability of each referent *P*(*r* ∣ *ρ*_*i*_). This prior probability is a function of the common ground *ρ* shared between speaker and listener in that interacting around the objects changes the probability that they will be referred to later. We assume that individuals vary in their sensitivity to common ground which, captured in participant-specific parameters *ρ*_*i*_. Note that this view ignores that there might be other aspects of a referent (such as perceptual salience or familiarity) that might influence the prior probability of it being the referent. While we do think that these aspects might matter, we tried to minimize their influence by way of carefully designing and selecting the stimuli used in the experiments.

To decide between referents, the listener (*L*_1_) reasons about what a rational speaker (*S*_1_) would say given an intended referent. This speaker is assumed to compute the informativity for each available utterance and then choose an utterance in proportion to its informativity raised to the power of the parameter *α*. As such, *α* reflects how informative the listener expects the speaker to be (with values above 1 speaking for a stronger expectation). This expectation may vary between individuals, leading to a participant-specific parameter *α*_*i*_:

$$P_{S_{1}}\left(u|r;\left\{\alpha_{i}\theta_{ij}\right\}\right)\propto P_{L_{0}}{\left(r|u;\left\{\theta_{ij}\right\}\right)}^{\alpha_{i}}$$ (2)

The informativity of each utterance is given by imagining which referent a literal listener (*L*_0_), who interprets words according to their lexicon 𝓛, would infer upon hearing the utterance. This reasoning depends on what kind of semantic knowledge (word–object mappings, *θ*) the speaker thinks the literal listener has. For familiar objects, we take semantic knowledge to be a function of the degree-of-acquisition of the associated word, which we assume to vary between individuals (*θ*_*ij*_).

$$P_{L_{0}}\left(r|u;\left\{\theta_{ij}\right\}\right)\propto 𝓛\left(u,r|\theta_{ij}\right)$$ (3)

This modeling framework describes how different information sources are integrated and how individuals might differ from one another. More specifically, we assume individual differences to arise from varying sensitivities to the three information sources (captured in the participant-specific parameters *ρ*_*i*_, *α*_*i*_, and *θ*_*i*,*j*_). The process by which information is integrated is thought to follow the same rational (Bayesian) procedure for all participants. Given participant-specific values for the three sensitivity parameters, this model allows us to generate participant-specific predictions for situations in which information needs to be integrated. Next, we describe how we estimated these participant-specific parameter values based on the data collected in Part 1.

#### Parameter Estimation.

Models to estimate parameters were implemented in the probabilistic programming language webppl (Goodman & Stuhlmüller, 2014). As noted above, the three information sources were: sensitivity to common ground (*ρ*_*i*_), expectations about speaker informativeness (*α*_*i*_), and semantic knowledge (*θ*_*ij*_). Figure 1 shows which tasks informed which parameters. All parameters were estimated via hierarchical regression (mixed-effects) models. That is, for each parameter, we estimated an intercept and slope (fixed effects) that best described the developmental trajectory for this parameter based on the available data. Participant-specific parameters values (random effects) were estimated as deviations from the value expected for a participant based on their age (standardized so that minimum age was 0). Details about the estimation procedure can be found in the supplementary material and code to run the models can be found in the associated online repository.

The parameters for semantic knowledge (*θ*_*ij*_) were simultaneously inferred from the data from the mutual exclusivity, the comprehension, and the production experiments. To leverage the mutual exclusivity data, we adapted the RSA model described above to a situation in which both objects (novel and familiar) had equal prior probability (i.e., no common ground information). In the same model, we also estimated the parameter for speaker informativeness (see below).

For the comprehension experiment, we assumed that the child knew the referent for the word with probability *θ*_*ij*_. If *θ*_*ij*_ indicated that they knew the referent (a coin with weight *θ*_*ij*_ comes up heads) they would select the correct picture; if not they would select the correct picture at a rate expected by chance (1/6). Likewise, for the production experiment, we assumed that the child knew the word for the referent with probability *θ*_*ij*_. If *θ*_*ij*_ indicated that they knew the word (a coin with weight *θ*_*ij*_ comes up heads), we assumed the child would be able to produce it with probability *γ*. This successful-production-probability *γ* was the same for all children and was inferred based on the data. This adjustment reflects the finding that children’s receptive vocabulary for nouns tends to be larger than the productive (Clark & Hecht, 1983; Frank et al., 2021). Taken together, for each child *i* and familiar object *j* there were three data points to inform *θ*: one trial from the mutual exclusivity, one from the comprehension and one from the production experiment.

As noted above, the participant- and object-specific parameter (*θ*_*ij*_) was estimated in the form of a hierarchical regression model: *θ*_*ij*_ = logistic($\beta_{0,j}^{\theta }$ + *i* · $\beta_{1,j}^{\theta }$); each word’s lexical development trajectory (the intercept $\beta_{0,j}^{\theta }$ and slope $\beta_{1,j}^{\theta }$ of the regression line for each object) was estimated as a deviation from an overall trajectory of vocabulary development. The intercept and slope for each item were sampled from Gaussian distributions with means $\mu_{0}^{\theta }$, $\mu_{1}^{\theta }$ and variances $\sigma_{0}^{\theta }$, $\sigma_{1}^{\theta }$: $\beta_{0,j}^{\theta }$ ∼ 𝒩($\mu_{0}^{\theta }$, $\sigma_{0}^{\theta }$) and $\beta_{1,j}^{\theta }$ ∼ 𝒩($\mu_{1}^{\theta }$, $\sigma_{1}^{\theta }$). $\mu_{0}^{\theta }$ and $\mu_{1}^{\theta }$ represented the overall vocabulary development independent of particular familiar word–object pairings, and $\sigma_{0}^{\theta }$ and $\sigma_{1}^{\theta }$ represented the overall variability of intercepts and of slopes between items.

The parameter representing a child’s expectations about how informative speakers are (*α*_*i*_), was estimated based on the data from the mutual exclusivity experiment. As mentioned above, this was done jointly with semantic knowledge in a RSA model adopted to a situation with equal prior probability of the two objects (novel and familiar). Thus, for each child, there were 16 data points to inform *α*.

To estimate the participant specific parameter, we used the same approach as for semantic knowledge. That is, *α*_*i*_ was estimated via a linear regression – *α*_*i*_ = $\beta_{0}^{\alpha }$ + *i* · $\beta_{1}^{\alpha }$ – in which $\beta_{0}^{\alpha }$ and $\beta_{1}^{\alpha }$ defined a general developmental trajectory. Again, we assumed that children might deviate from their expectations about speaker informativeness based on their numerical age and so we estimated *i* as a deviation from the child’s numerical age *k*: *i* ∼ 𝒩(*k*, $\sigma_{i}^{\alpha }$).

We estimated children’s sensitivity to common ground (*ρ*_*i*_) based on the 12 data points from the discourse novelty experiment. We used a logistic regression model to estimate the average developmental trajectory: *ρ*_*i*_ = logistic($\beta_{0}^{\rho }$ + *i* · $\beta_{1}^{\rho }$). To generate participant specific values for *ρ* we again estimated *i* as a deviation from the child’s numerical age *k*: *i* ∼ 𝒩(*k*, $\sigma_{i}^{\rho }$).

### Results

**Figure 2.**
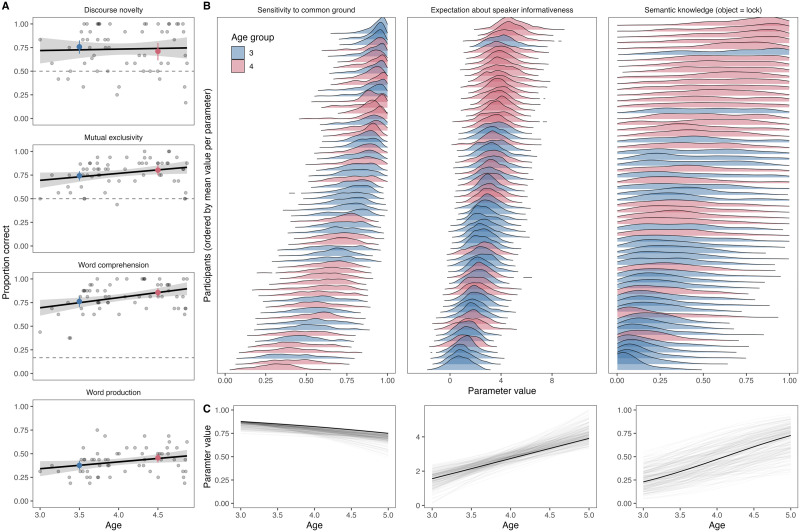
**Results for the sensitivity tasks.** (A) Proportion of correct responses in each task by age. Colored dots show the mean proportion of correct responses (with 95% CI) binned by year. Regression lines show fitted generalized linear models with 95% CIs. (B) Posterior distributions for each parameter (information source) and participant, ordered by mean value, separate for each parameter. Color shows age group. (C) Average developmental trajectories for the three sensitivity parameters based on the hyper-parameters extracted from the model.

Figure 2 visualizes the results for the four sensitivity tasks and the participant-specific model parameters estimated from the data. In all four tasks, we saw that children performed above chance (not applicable in the case of word production), suggesting that they made the alleged pragmatic inference or knew (some) of the words for the objects involved. With respect to age, performance in raw test scores seemed to increase with age in the three tasks relying on semantic knowledge (mutual exclusivity, word production and word comprehension). Performance in these tasks was also correlated (see supplementary material). For discourse novelty, performance did not increase with age.

The hierarchical nature of the parameter estimation procedure in our model allowed us to take an aggregate look at these results in what they indicate about the development of sensitivity to the different information sources. For this, we extracted the posterior distributions for intercepts and slopes for the parameter estimates corresponding to the different information sources (*α*, *ρ*, and *θ*) based on which the participant-specific estimates were sampled. These values can be taken to describe the average developmental trajectory for the respective parameter and with that, the sensitivity to the respective information source. For expectations about speaker informativeness, the intercept was larger than 1 (mode = 1.56; 95% HDI = 0.66–2.38) and the slope was positive (mode = 1.18; 95% HDI = 0.73–2.12) suggesting that already the youngest children (age was standardized so that minimum age was 0) were expecting the speaker to be informative and this expectation increased with age. For sensitivity to common ground, the intercept was positive (mode = 1.96; 95% HDI = 1.32–2) while the slope was negative (mode = −0.43; 95% HDI = −0.84 – −0.17) showing that sensitivity to common ground was very high at 3 years of age (probability to select the discourse-novel object = *logistic* (1.96) = 0.88) and slightly decreased with age. For semantic knowledge, the intercept and slope represent the overall vocabulary development independent of particular familiar word–object pairings (conditional on the familiar objects involved in the study). At 3 years of age, the average probability to know the label for a word was 0.23 (*logistic* (−1.21); intercept estimate: mode = −1.21; 95% HDI = −2.47–0.01), which substantially increased with age (slope estimate: mode = 1.10; 95% HDI = 0.28–1.83). To contextualize the semantic knowledge of the different familiar objects, we correlated the probability to know a word (averaged across participants) with age-of-acquisition ratings for English translations these words obtained by Kuperman et al. (2012)^1^. We found a strong negative correlation of *r* = −0.59, suggesting that participants (German children) had less semantic knowledge of words that were rated (by adult English-speakers) to be acquired later in development.

Most importantly, however, we saw considerable variation in raw scores between individuals (see Figure 2). When focusing on the participant-specific parameter estimates (Figure 2B), we saw that parameters that were estimated based on more data (sensitivity to common ground – 12 trials, and expectations about speaker informativeness – 16 trials) had better defined posterior distributions in comparison to the semantic knowledge parameters, which were based on fewer data (3 trials per object).

### Discussion

In Part 1, we estimated participant-specific parameters representing each individual’s sensitivity to the three information sources. We found that, as a group, children were sensitive to the different information sources we measured. Furthermore, there was substantial variation between individuals in *how* sensitive they were to each information source. These results provided a solid basis for studying information integration in Part 2.

## PART 2: INTEGRATION

### Methods

The study was pre-registered and all data, analysis script and materials are publicly available (see Part 1 for more information).

#### Participants.

Participants were the same as in Part 1.

#### Procedure.

The task was implemented in the same environment as the tasks in Part 1. Each child completed the combination task in the second testing session. The general procedure followed that of the discourse novelty task, however, only one of the objects was unknown while the other was familiar. The combination task had two conditions. In the *congruent condition*, the unfamiliar object was also new to discourse. For example, at the beginning of the trial, a familiar object (e.g., a lock) was on one table while the other table was empty. When the agent disappeared, a novel object appeared. When the experimenter returned and used a novel nonce-word both the mutual exclusivity and discourse inferences pointed to the novel object as the referent of the novel word (see also Figure 1). In the *incongruent condition*, the familiar object was new to discourse and thus the two inferences pointed to different objects (the mutual exclusivity inference would suggest the novel object but the common ground would suggest the familiar object). The idea behind having these different conditions was to increase variability in children’s responses to test the scope of the model. We created matched pairs for the 16 familiar objects and assigned one object of each pair to one of the two conditions. Thus, there were eight trials per condition in the combination task in which each trial was with a different familiar object. We counterbalanced the order of conditions and the side on which the discourse-novel object appeared. Responses were coded from a mutual exclusivity perspective (choosing novel object = 1). All children received the same order of trials. There was the option to terminate the study after 8 trials (two children).

### Analysis

We used the rational integration model described above to generate predictions for each participant and trial in the combination task based on the participant-specific parameters estimated in Part 1. That is, for each combination of *ρ*, *α*, and *θ* for participant *i* and familiar object *j*, the model returned a distribution for the probability with which the child should choose the novel object.

We contrasted the predictions made by the rational integration model described above to those made by two plausible alternative models which assume that children selectively ignore some of the available information sources (Gagliardi et al., 2017). These models generated predictions based on the same parameters as the *rational integration* model, the only difference lay in how the parameters were used.

The *no speaker informativeness* model assumed that the speaker does not communicate in an informative way. This corresponds to *α*_*i*_ = 0, which causes the likelihood term to always be 1. As a consequence, this model also ignores semantic knowledge (which affects the likelihood term) and the predictions of this model correspond to the prior distribution over objects:

$$P_{L_{1}}^{\text{no\_si}}\left(r|u;\left\{\rho_{i}\right\}\right)\propto P\left(r|\rho_{i}\right)$$ (4)

On the other hand, the *no common ground* model ignores common ground information, *ρ*_*i*_. This model takes in object-specific semantic knowledge and speaker informativeness but uses a prior distribution over objects that is constant across alignment conditions and uniform (e.g., [0.5, 0.5]). This model corresponds to a listener who only focuses on the mutual exclusivity inference and ignores the common ground manipulation. As a consequence, the listener does not differentiate between the two common ground alignment conditions.

$$P_{L_{1}}^{\text{no\_cg}}\left(r|u;\left\{\alpha_{i}\theta_{ij}\right\}\right)\propto P_{S_{1}}\left(u|r;\left\{\alpha_{i},\theta_{ij}\right\}\right)$$ (5)

We evaluated the model predictions in two steps. First, we replicated the group-level results of Bohn et al. (2021). That is, we compared the three models in how well they predicted the data of the combination task when aggregated across individuals. For this, we correlated model predictions and the data (aggregated by trial and age group) and computed Bayes Factors comparing models based on the marginal likelihood of the data given the model.

Second, and most importantly, we evaluated how well the model predicted performance on an *individual* level. For each trial, we converted the (continuous) probability distribution returned by the model into a binary prediction (the structure of the data) by flipping a coin with the Maximum a posteriori estimate (MAP) of the distribution as its weight^2^. For the focal and the two alternative models, we then computed the proportion of trials for which the model predictions matched children’s responses and compared them to a level expected by random guessing using a Bayesian t-test. Finally, for each child, we computed the Bayes Factor in favor of the *rational integration* model and checked for how many children this value was above 1 (log-Bayes Factors > 0). Bayes Factors larger than 1 present evidence in favor of the *rational integration* model. We evaluated the distribution of Bayes Factors following the classification of Lee and Wagenmakers (2014).

### Results

**Figure 3.**
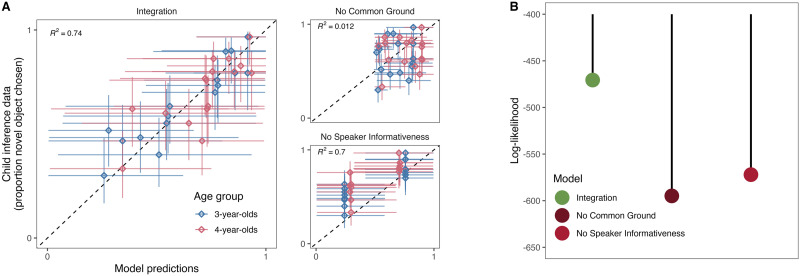
**Group-level model comparison.** (A) Correlation between model predictions and data (aggregated across individuals and binned by year with 95% HDI) for each trial in the combination experiment. (B) Log-likelihood for each model given the data.

On a group-level, the results of the present study replicated those of Bohn et al. (2021). The predictions made by the rational integration model were highly correlated with children’s responses in the combination task. The model explained around 74% of the variance in the data and with that more compared to the two alternative models (Figure 3A). Bayes Factors computed via the marginal likelihood of the data (Figure 3B) strongly favored the *rational integration* model in comparison to the *no common ground* (*BF*_10_ = 9.1e+53) as well as the *no speaker informativeness* model (*BF*_10_ = 1.2e+44).

**Figure 4.**
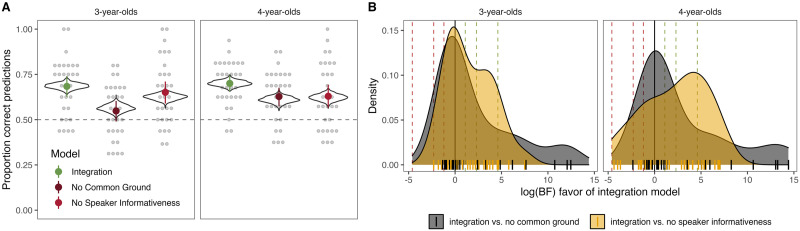
**Individual-level model comparison.** (A) Proportion of correct predictions for each model. Solid colored dots show mean with 95% CI for one run of the coin flip procedure. Light dots show aggregated individual data for the same run. Violins show the distribution of means for 1000 runs of the procedure. (B) Distribution of log-Bayes Factors for each individual. Dashed lines show Bayes Factor thresholds of 3, 10 and 100.

Next, we turned to the individual-level results. When looking at the proportion of correct predictions (for one run of the coin-flipping procedure), we saw that the *rational integration* model correctly predicted children’s responses in the combination task in 72% of trials, which was well above chance (*BF*_10_ = 2.15e+14) and numerically higher compared to the two alternative models (Figure 4A). Note that the alternative models also predicted children’s responses at a level above chance (*no common ground*: 61%, *BF*_10_ = 220251; *no speaker informativeness*: 60%, *BF*_10_ = 55.4), emphasizing that they constitute plausible alternatives. In the supplementary material we also compared models with respect to the situations in which they did or did not correctly predict children’s responses.

When directly comparing the models on an individual level, we found that the *rational integration* model provided the best fit for the majority of children. In comparison to the *no common ground* model, 62% of Bayes Factors were larger than 1 and 35% were larger than 10. In comparison to the *no speaker informativeness* model, 68% of Bayes Factors were larger than 1 and 45% were larger than 10 (Figure 4B).

### Discussion

The results of Part 2 show that the *rational integration* model accurately predicted children’s responses in the combination task. Importantly, this was the case not just on a group level, but also on an individual level where the model correctly predicted children’s responses in the majority of trials. Furthermore, it was more likely to be correct and provided a better explanation of the data compared to two alternative models that assumed that children selectively ignored some of the information sources.

## GENERAL DISCUSSION

Probabilistic models of cognition are often used to describe human performance in the aggregate, but these successes do not necessarily imply that they correctly describe individuals’ judgments. Instead, individual judgments could be produced via the operation of simpler heuristics. We investigated this study using rational speech act models of children’s pragmatic reasoning as a case study, using a computational cognitive model to make out-of-sample predictions about individual children’s behavior on a trial-by-trial basis. In Part 1, we used data from four tasks to estimate child-specific sensitivity parameters capturing their semantic knowledge, expectations about speaker informativeness, and sensitivity to common ground. In Part 2, we used these parameters to predict how the same children should behave in a new task in which all three information sources were jointly manipulated. We found strong support for our focal *rational integration* model in that this model accurately predicted children’s responses in the majority of trials and provided a better fit to individuals’ performance compared to two alternative heuristic models. Taken together, this work provides a strong test of the theoretical assumptions built into the model and both replicates and extends prior research that showed pragmatic cue integration in children’s word learning in the aggregate (Bohn et al., 2021).

The *rational integration* model was built around three main theoretical assumptions. First, it assumes that children integrate all available information sources. The model comparison, in which we compared the focal model to two models that selectively ignored some of the information sources, strongly supported this assumption. For the majority of individuals – as well as on a group level – this model provided the best fit. Zooming out, this result strengthens the assumption that language learning and comprehension are social inferences processes during which listeners integrate different information sources to infer the speaker’s intention (Bohn & Frank, 2019; Clark, 2009; Tomasello, 2009). At any given moment, different pathways may lead to the same goal, and the lack of one type of information source might be compensated by the availability of another. This view highlights the resilience of human communicative abilities.

However, for some individuals, one of the alternative models provided a better fit. Many of the Bayes Factors in these cases were relatively close to zero, but in a few cases, there was substantial evidence for the alternative models. Finding out why this is the case and what characterizes these individuals (e.g. if support for a lesioned model can be linked to other psychological constructs like attention or memory abilities) would be an interesting avenue for future research.

The second assumption built into the model is that the integration process does not change with age. We did not probe this assumption in the present study because, in order to do so on an individual level, it would require longitudinal data – an interesting extension for future work. Finally, the model assumes that children differ in their sensitivity to the different information sources but *not* in the way they integrate information. Even though a model using this assumption predicted the data well, it would also be interesting to explore structural differences between individuals. For example, Franke and Degen (2016) conceptualized individual differences in pragmatic reasoning in terms of mind-reading abilities or “depth of recursion” (Camerer et al., 2004). In modeling terms, this corresponded to adding additional layers of speakers and listeners to the RSA model. This approach implies that individual differences are qualitative (i.e., individuals engage in qualitatively different reasoning processes) and not merely quantitative as in the model presented here. It would be interesting for future research to identify situations in which these two approaches could be directly compared to one another (see Rouder & Haaf, 2021 for a discussion of quantitative vs. qualitative individual differences).

Although our model explains and predicts data, we should be careful with granting the processes and parameters in it too much psychological realism. Nevertheless, we think that when studying individual differences, the model parameters can be interpreted as candidate latent measures of the psychological processes – this interpretation is not necessarily worse than using raw performance scores as a description of individuals (Borsboom, 2006).

In further support of the idea that model parameters can capture individual variation, our model parameters are estimated by taking into account the structure and the different processes involved in the task. This estimation process means that individual parameters can be based on data from multiple tasks, as, for example, semantic knowledge was estimated based on the mutual exclusivity, comprehension and production tasks. Support for such an approach comes from a recent study that used an RSA-type model to estimate a single parameter that captured children’s pragmatic abilities based on data from three tasks (Bohn et al., 2022a, 2022b). Taken together we think that computational modeling can make an important contribution to studying individual differences on a process level.

Our study is limited in terms of generalizability because we tested only one sample of children growing up in a western, affluent setting. However, the modeling approach put forward here provides an interesting way of studying and theorizing about cross-cultural differences. Following Bohn and Frank (2019), our prima facie assumption is that children from different cultural settings might differ in terms of their sensitivity to different information sources – just like individuals differ within cultural settings – but the way that information is integrated is hypothesized to be the same across cultures. This prediction could be tested by comparing alternative models that make different assumptions about the integration process.

In sum, we have shown that children’s pragmatic word learning can be predicted on a trial-by-trial basis by a computational cognitive model. Together with previous work that focused on aggregated developmental trajectories (Bohn et al., 2021), these findings suggest that the same computational processes – a pragmatic inference process that integrates sources of information in a rational manner – can be used to predict group- and individual-level data.

## AUTHOR CONTRIBUTIONS

Manuel Bohn: Conceptualization, Formal analysis, Methodology, Visualization, Writing – original draft, Writing – review & editing. Louisa S. Schmidt: Conceptualization, Investigation, Methodology, Writing – original draft, Writing – review & editing. Cornelia Schulze: Conceptualization, Methodology, Writing – review & editing. Michael C. Frank: Conceptualization, Writing – review & editing. Michael Henry Tessler: Conceptualization, Formal analysis, Methodology, Writing – review & editing.

## FUNDING INFORMATION

M. H. Tessler was funded by the National Science Foundation SBE Postdoctoral Research Fellowship Grant No. 1911790. M. C. Frank was supported by a Jacobs Foundation Advanced Research Fellowship and the Zhou Fund for Language and Cognition. The funders had no role in study design, data collection and analysis, decision to publish, or preparation of the manuscript.

## Notes

^1^ German ratings were not available for all words.^2^ Note that this procedure is not deterministic and the results will slightly vary from one execution to the next (see also Figure 4).

## Supplemental Materials

https://osf.io/pa5x2

https://github.com/manuelbohn/spin-within
