# Supplementary material for: Modeling Individual Differences in Children’s Information Integration During Pragmatic Word Learning
Source: Open Mind (Camb). 2022 Dec 16;6:311–26. doi: 10.1162/opmi_a_00069 (PMC10042310; doi:10.1162/opmi_a_00069)
Supplement: Supplementary file 1 [file opmi-06-311-s001.pdf]

Modeling individual differences in children's information integration during pragmatic word learning

Supplementary material

Manuel Bohn, Louisa S. Schmidt, Cornelia Schulze, Michael C. Frank and Michael Henry Tessler

Contents

Overview **1**

Empirical studies **2**

Sample size . . . 2

Sensitivity experiments . . . 2

Combination experiment . . . 5

Cognitive models **5**

Modelling framework . . . 5

Model parameters . . . 7

Model predicitons . . . 9

Model evaluation . . . 9

Participant-specific model predictions . . . 11

Appendix 1: Group model **13**

Comparison ID vs. Group model . . . 13

Appendix 2: Model parameters **13**

References **16**

Overview

The goal of the study was to predict information integration during pragmatic word learning in children on a trial-by-trial basis. We measured children's sensitivity to different information sources and then used an RSA model to generate how the same children should behave when these information sources need to be integrated.

Table 1: Sample size and demographic information.

Age group	Sex	N
3	f	15
3	m	15
4	f	15
4	m	15

Empirical studies

Sample size

Table 1 gives an overview of the participants. All 60 participants participated in all tasks (4 tasks to measure sensitivity to information sources and the combination task).

Sensitivity experiments

Setup

The different tasks were programmed as interactive picture books in `JavaScript/HTML` and presented on a website. During the video call (via BigBlueButton), participants would enter the website with the different tasks and share their screen. The experimenter guided them through the procedure and told caregivers when to advance to the next task. Children responded by pointing to objects on the screen, which their caregivers would then select for them via mouse click. For the production task, the experimenter shared their screen and presented pictures in a slide show. For the mutual exclusivity, discourse novelty, and combination tasks, pre-recorded sound files were used to address the child.

Tasks

We used the same tasks as in Bohn et al. (2021) to measure children's sensitivity to the different information sources, that is:

- Mutual Exclusivity
- Discourse novelty

In addition, we used two new vocabulary tasks:

- Word production
- Word comprehension

Across all tasks (except discourse novelty) we used a total of 16 familiar objects. There was one trial for each familiar object in mutual exclusivity, word production and comprehension. For the 16 familiar objects, the level of familiarity varied. Four objects (duck, bread, carrot, apple) were retained from Bohn et al. (2021) and can be considered highly familiar objects for 3- and 4-year-old children. Twelve additional objects were modeled after a previous mutual exclusivity study involving German 3- and 4-year-olds Grassmann, Schulze, and Tomasello (2015). Some of these objects (e.g., corkscrew) are barely familiar for children in this age group.

Training Generally, each child participated in two sessions. Both sessions began with a brief training. First, the child was instructed to point to colorful dots on the screen, which disappeared when their parent selected them. This way the child could practice pointing at the screen and the parent could practice interpreting their child's pointing gestures. Next, there were two training trials where animal speakers requested highly familiar objects (duck and teddy bear) from the child. This introduced children to the procedure of the following tasks, where animals requested objects from them and they had to point to the requested object.

Mutual exclusivity experiment Each child participated in the mutual exclusivity experiment (16 trials) on the first testing day. Here, animal speakers stood on a small hill between two tables on which objects were placed. One of these objects was potentially familiar to the child, the other object was unknown. After introducing themselves, speakers requested objects from the child using novel labels label (e.g., "Oh, cool, da liegt ein Höfas auf dem Tisch, wie toll! Ein Höfas liegt auf dem Tisch! Kannst du mir das Höfas geben?" ["Oh, cool, there is a höfas on the table, how neat! A höfas is on the table. Can you give me the höfas?"]).

The mutual exclusivity experiment had only one condition. Each familiar and unknown object appeared once; each speaker appeared four times. The dependent variable was the object the child selected on each trial (i.e., choice). Here, choosing the unknown object constituted the correct choice and was coded as 1.

Discourse novelty experiment Each child participated in the discourse novelty experiment (12 trials) on the first testing day, immediately following the mutual exclusivity experiment. Animal speakers again stood on a small hill between two tables. Contrary to the mutual exclusivity experiment, in the beginning, only one of the tables contained an object, while the other was empty. Speakers commented on the presence of the object ("Aha, schau mal da!" ["Aha, look at that!"]) and the absence of an object on the other table ("Hm, da ist nichts!" ["Hm, nothing there!"]). These utterances were designed to create common ground between the speaker and the child. Both the side where the empty table stood (left or right) and which of these comments the speaker uttered first was counterbalanced across trials. Speakers disappeared from the screen after the sound of a ringing telephone could be heard. (The child had been told beforehand that at times, the animals would have to leave to answer calls.) During the speakers' absence, a second object appeared on the previously empty table. Shortly after, the speaker returned and requested an object in the same manner as in the mutual exclusivity experiment (e.g., "Oh, toll, da liegt ein Wisslo auf dem Tisch, wie interessant! Ein Wisslo liegt auf dem Tisch! Kannst du mir das Wisslo geben?" ["Oh, neat, there's a wisslo on the table, how interesting! A wisslo is on the table. Can you give me the wisslo?"]).

The discourse novelty experiment had only one condition. Each unknown object appeared once; each speaker appeared three times. Again, the dependent variable was choice. Here, choosing the object that appeared later during the trial and was thus new in the discourse context constituted the correct choice and was coded as 1.

Combination experiment Each child participated in the combination experiment (16 trials) on the second testing day. Here, mutual exclusivity and discourse novelty were combined. Overall, the procedure was the same as in the discourse novelty experiment. However, in contrast to the discourse novelty experiment, only one of the object was unknown, while the other object was familiar. Speakers requested objects in the same manner as before.

The combination experiment had two conditions. In the congruent condition, the object that was new in the discourse context was an unknown object, that is, the pragmatic cues mutual exclusivity and discourse novelty were aligned. In the incongruent condition, the object that was new in the discourse context was a familiar object, that is, the pragmatic cues were disaligned.

Objects with comparable familiarity were assigned to the conditions (e.g., children are similarly familiar with duck and carrot; duck was assigned to the congruent condition and carrot was assigned to the incongruent condition). The combination experiment consisted of 8 congruent and 8 incongruent trials (4 congruent and

4 incongruent trials when the procedure was stopped early). For our coding, choosing the unknown object constituted the correct choice and was coded as 1.

For all three tasks, children received the same order of trials to ensure comparability across participants.

Reliability Coding Reliability coding was performed to ensure that the objects parents selected matched the objects children had chosen via pointing in each respective trial. For a randomly selected 20% of the sample (n = 12), children's object choices were coded from video by a coder who was blind to the data from parents' selections. The results indicate that parents faithfully selected children's chosen objects (99.74% match, κ= .997, $p < .001$).

Word production task The word production and the comprehension task were run on the second testing day after the mutual exclusivity, the discourse novelty and the combination experiment.

For the production task, the experimenter showed the child an image of an object (the same as was used in the other experiments) and prompted them to label the object with probing questions such as: "Weißt du, wie man das hier nennt?" ["Do you know what this is called?"] or "Was ist das?" ["What's this?"]. The trial was coded as correct and received a score of 1 if the child produced a label that was correct according to coding scheme that had been developed prior to data collection based on piloting data. Otherwise, the child's answer received a score of 0. The experimenter went through each of the 16 objects one by one. the coding scheme is available in the associated repository at `documentation/production_task_list_of_labels.pdf`.

The picture below shows an example.The images were the same as the ones used in the mutual exclusivity task and the comprehension task.

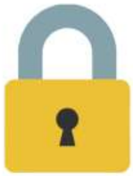

Figure 1: Example stimulus for the production task.

Comprehension task The child was shown images of 6 objects on a screen, 4 of which were objects that appeared in the rest of the study and 2 were distractors. The experimenter asked the child to pick out an object (e.g., "Wo ist der Korkenzieher?" ["Where is the corkscrew?"] or "Kannst du auf das Schloss zeigen?" ["Can you point to the lock?"]). The child responded by pointing at an object, which the parent then selected.

We coded as correct if the child selected the correct object OR if they had correctly named the object during production.

The experimenter went through all 16 familiar objects on a total of 4 slides (each showing 6 objects, including 2 distractors).

The picture below shows an example (familiar objects from the study: rasp, lock, apple, hanger; distractors: dog toy and hydrant).

Results

Figure 3A shows the results for the four sensitivity experiments designed to measure children's sensitivity to individual information sources. In all cases, children of all ages children were sensitive to the respective

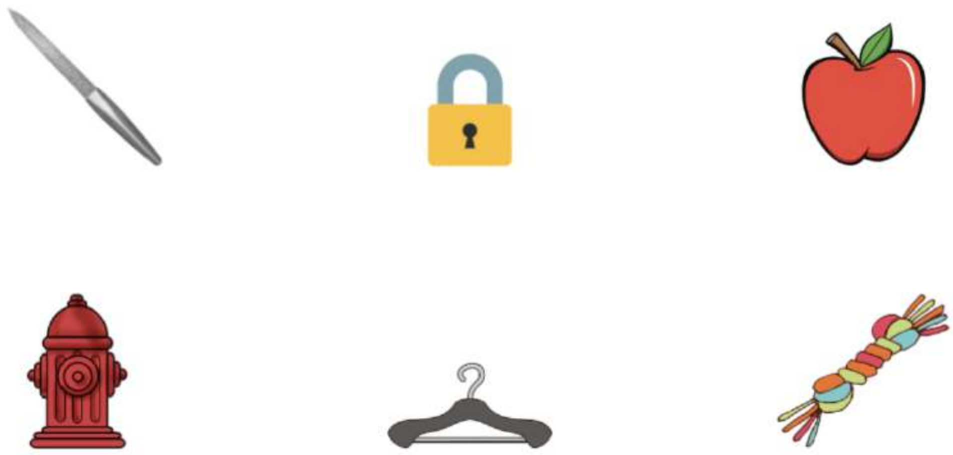

Figure 2: Example for layout of a trial in the comprehension task.

information sources (i.e. performance above chance or above 0) For word comprehension and production and mutual exclusivity we see an increase of performance with age whereas for discourse novelty, performance was stable across development.

Furthermore, the three tasks measuring children's semantic knowledge were positively correlated (Figure 3B). However, the correlation was not perfect, suggesting that the tasks were not redundant.

Combination experiment

There were also 16 trials in combination, half of which were in the congruent condition and the other half were in the incongruent condition. The familiar objects from the mutual exclusivity experiment were therefore split across the two conditions.

The combination of condition (congruent/incongruent) and familiar object was the same for all children and so was the order of trials and side counterbalancing.

Results

Since there is no clear right or wrong answer it is difficult to evaluate the combination experiment on its own. Figure 4 below gives some kind of sanity check, namely that in the congruent case, children should be more likely to choose the unfamiliar object compared to the mutual exclusivity experiment whereas in the incongruent condition they should be less likely to do so. This is generally what we see.

Cognitive models

Modelling framework

We adopted the modelling framework used by Bohn et al. (2021). A detailed description of that framework can be found in their supplementary material (click here for direct access to this document). In short, our models are situated in the Rational Speech Act (RSA) framework (Frank and Goodman 2012; Goodman and Frank 2016). RSA models are models of pragmatic reasoning and treat language understanding as a special case of Bayesian social reasoning. A listener interprets an utterance by assuming it was produced

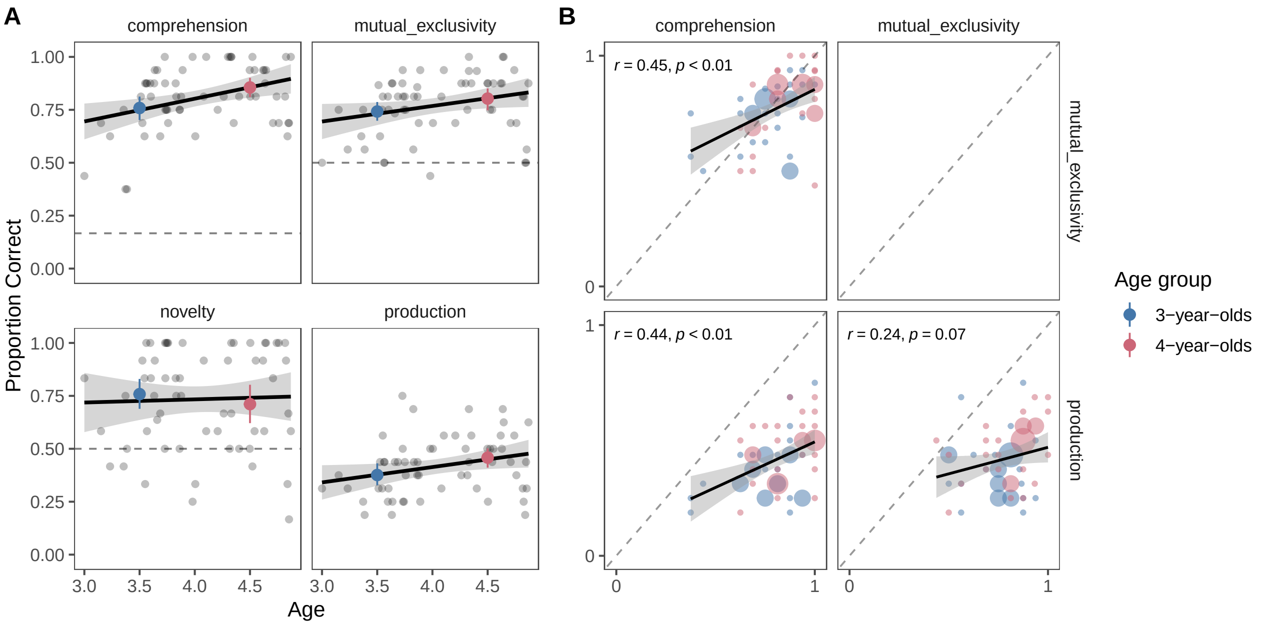

Figure 3: A) Results of individual experiments. Colored points show means (with 95% CI) for data binned by year, light black dots show participant means. Dotted line shows level of performance expected by chance. B) Correlation between the three tasks measuring semantic knowledge.

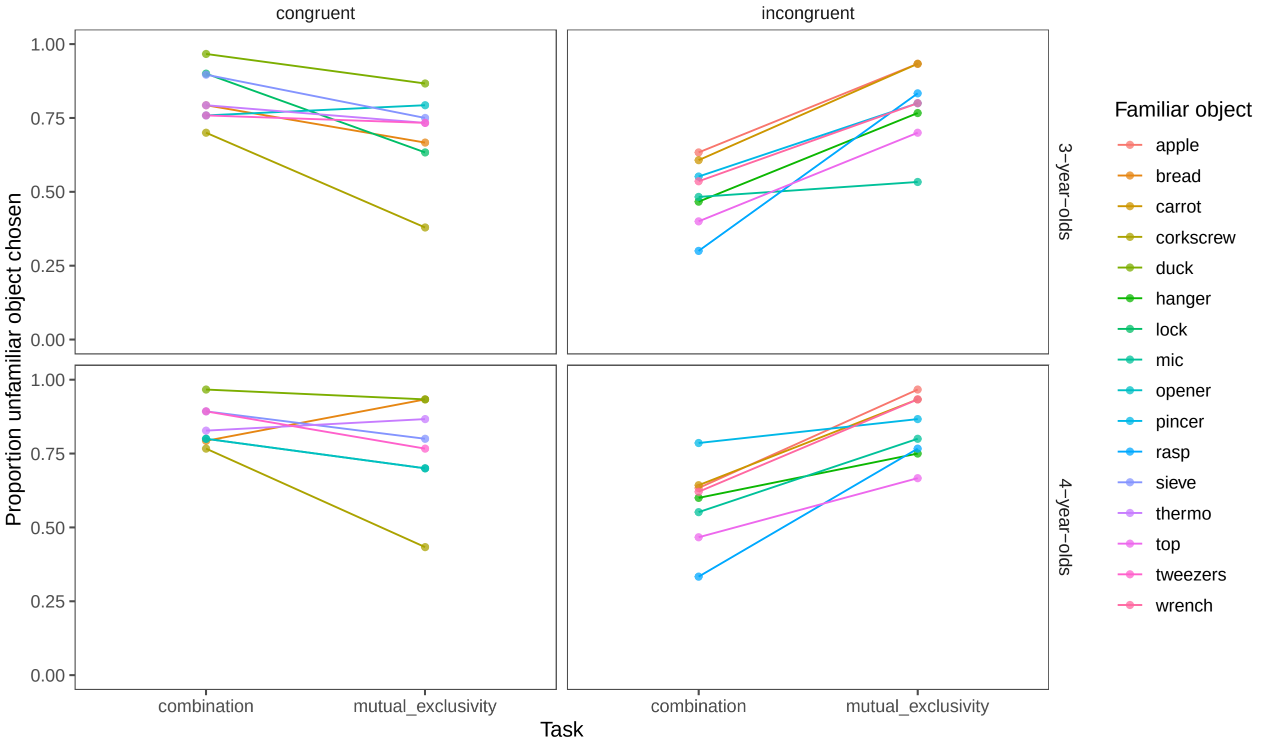

Figure 4: Average proportion with which the unfamiliar object was chosen in the mutual exclusivity task and the combination experiment depending on the familiar object. Congruent and incongruent refers to the condition in which the familiar object appeared in the combination experiment.

by a cooperative speaker who had the goal to be informative. Being informative is defined as providing messages that increase the probability of the listener inferring the speaker's intended message. The *rational integration* model, including all data-analytic parameters, is formally defined as:

$$P_{L_1}(r \mid u; \{\rho_i, \alpha_i\, \theta_{ij}\}) \propto P_{S_1}(u \mid r; \{\alpha_i, \theta_{ij}\}) \cdot P(r \mid \rho_i) \tag{1}$$

$$P_{S_1}(u \mid r; \{\alpha_i\, \theta_{ij}\}) \propto P_{L_0}(r \mid u; \{\theta_{ij}\})^{\alpha_i} \tag{2}$$

$$P_{L_0}(r \mid u; \{\theta_{ij}\}) \propto \mathcal{L}(u, r \mid \theta_{ij}) \tag{3}$$

Thus, the model describes a listener (L_1) reasoning about the intended referent of a speaker's (S_1) utterance. This reasoning is contextualized by the prior probability of each referent $P(r \mid \rho_i)$. This prior probability is a function of the common ground ρ shared between speaker and listener in that interacting around the objects changes the probability that they will be referred to later. We assume that children differ in their sensitivity to common ground and each child i is therefore represented by a different parameter value.

To decide between referents, the listener (L_1) reasons about what a rational speaker (S_1) would say given an intended referent. This speaker is assumed to compute the informativity for each available utterance and then choose the most informative one. The expectation of speaker informativeness may vary and is captured by the parameter α. Again, we assume that children differ in their expectations about how informative the speaker and each child i is therefore represented by a different value of α.

The informativity of each utterance is given by imagining which referent a literal listener (L_0), who interprets words according to their lexicon $\mathcal{L}$, would infer upon hearing the utterance. This reasoning depends on what kind of semantic knowledge (word–object mappings) the speaker thinks the literal listener has. We parameterize the listener's knowledge of a word's semantics in terms of a semantic knowledge parameter θ, which varies between 0 and 1. $\theta = 0$ corresponds to the state of knowledge for a completely novel word and results in a semantic interpretation function that chooses randomly between the objects in the scene. For each of the novel words, the literal listener is assumed to have semantic knowledge of 0. For $\theta \in (0, 1)$, the semantic interpretation function will select the familiar referent with probability $\theta + (1-\theta)\frac{1}{2} = \frac{1+\theta}{2}$; that is, with probability θ, the listener knows the correct meaning of the word (and picks out the correct referent 100% of the time); with probability $1-\theta$, the listener does not know the meaning of the word and must guess, picking out the correct referent 50% of the time. For familiar objects, we take semantic knowledge to be a function of the degree-of-acquisition of the associated word. We assume that children differ in their semantic knowledge fot the different familiar objects and θ therefore depends on the word j and the child i.

Model parameters

As mentioned above, each child in our model is represented by their own set of data-analytic parameters (ρ_i,α_i, and θ_{ij}). This will allow us to later use the model to generate participant-specific model predictions for the integration experiment. We estimated these parameters based on four sensitivity experiments. All parameters were estimated via hierarchical regression (mixed-effects) models. For each parameter, we estimated an intercept and slope (fixed effects) that best described the developmental trajectory for this parameter based on the available data. Participant-specific parameters values (random effects) were estimated as deviations from the value expected for a participant based on their age.

Semantic knowledge

The parameters for semantic knowledge were simultaneously informed by the data from the mutual exclusivity, the comprehension and the production experiments. To leverage the mutual exclusivity data, we adopted the RSA model described above to a situation in which both objects (novel and familiar) had equal prior

probability (i.e., no common ground information). In the same model, we also estimated the parameter for speaker informativeness (see below). For the comprehension experiment, we assumed that the child knew the referent for the word with probability θ_{ij}. If θ_{ij} indicated that they knew the referent (a coin with weight θ_{ij} comes up heads) they would select the correct picture; if not they would select the correct picture at a rate expected by chance (1/6). Likewise, for the production experiment, we assumed that the child knew the word for the referent with probability θ_{ij}. If θ_{ij} indicated that they knew the word (a coin with weight θ_{ij} comes up heads), we assumed the child would be able to produce it with probability γ. This successful-production-probability γ was the same for all children and was inferred based on the data. This adjustment reflects the finding that children's receptive vocabulary is larger than the productive. Taken together, for each child i and familiar object j there were three data points to inform θ: one trial from the mutual exclusivity, one from the comprehension and one from the production experiment.

The participant- and object-specific parameter (θ_{ij}) was estimated in the form of a hierarchical regression model: $\theta_{ij} = \text{logistic}(\beta^{\theta}_{0,j} + i \cdot \beta^{\theta}_{1,j})$; each word's lexical development trajectory (the intercept $\beta^{\theta}_{0,j}$ and slope $\beta^{\theta}_{1,j}$ of the regression line for each object) was estimated as a deviation from an overall trajectory of vocabulary development. The intercept and slope for each item were sampled from Gaussian distributions with means $\mu^{\theta}_0, \mu^{\theta}_1$ and variances $\sigma^{\theta}_0, \sigma^{\theta}_1$: $\beta^{\theta}_{0,j} \sim \mathcal{N}(\mu^{\theta}_0, \sigma^{\theta}_0)$ and $\beta^{\theta}_{1,j} \sim \mathcal{N}(\mu^{\theta}_1, \sigma^{\theta}_1)$. μ^{θ}_0 and μ^{θ}_1 represented the overall vocabulary development independent of particular familiar word–object pairings, and σ^{θ}_0 and σ^{θ}_1 represented the overall variability of intercepts and of slopes between items (see Figure **??**).

In this model, the participant-specific value for θ was generated via i. Given the structure of the model, it is natural to think of i as the child's age. However, we took i to be the child's "linguistic" age. That is, we assumed that a child's semantic knowledge might be higher (or lower) than what we would expect given the child's numerical age. Thus, we sampled i from a Gaussian distribution with mean k and variance σ^{θ}_i: $i \sim \mathcal{N}(k, \sigma^{\theta}_i)$. Here k was the child's numerical age and σ^{θ}_i the overall variability in linguistic age between children. This procedure allowed us to inform the participant specific value for θ both by the child's responses but also by the overall developmental trajectory in the data.

Expectations about speaker informativeness

The parameter representing a child's expectations about how informative speakers are, was estimated based on the data from the mutual exclusivity experiment. As mentioned above, this was done jointly with semantic knowledge in a RSA model adopted to a situation with equal prior probability of the two objects (novel and familiar).

To estimate the participant specific parameter, we used the same approach as for semantic knowledge. That is, α_i was estimated via a linear regression – $\alpha_i = \beta^{\alpha}_0 + i \cdot \beta^{\alpha}_1$ – in which β^{α}_0 and β^{α}_1 defined a general developmental trajectory. Again, we assumed that children might deviate from their expectations about speaker informativeness based on their numerical age and so we estimated i as a deviation from the child's numerical age k: $i \sim \mathcal{N}(k, \sigma^{\alpha}_i)$.

Sensitivity to common ground

We estimated children's sensitivity to common ground based on the data from the discourse novelty experiment. We used a logisitc regression model to estimate the average developmental trajectory: $\rho_i = \text{logistic}(\beta^{\rho}_0 + i \cdot \beta^{\rho}_1)$. To generate participant specific values for ρ we again estimated i as a deviation from the child's numerical age k: $i \sim \mathcal{N}(k, \sigma^{\rho}_i)$.

Parameter estimation

All cognitive models and regression models were implemented in the probabilistic programming language `WebPPL` (Goodman and Stuhlmüller 2014). The corresponding model code can be found in the associated

online repository (file `model/spin-within_model_prediction.wppl`). We used the following prior distributions for model parameters. Intercept and slope for sensitivity to common ground: $\beta_0^\rho, \beta_1^\rho \sim \text{Uniform}(-2, 2)$. Variation between participants: $\sigma_i^\rho \sim \text{Uniform}(0, 4)$.

Speaker informativeness: $\beta_0^\alpha \sim \text{Uniform}(-3, 3)$ for the intercept and $\beta_1^\alpha \sim \text{Uniform}(-0, 4)$ for the slope. We restricted the slope to be positive because negative values for speaker informativeness are conceptually implausible. We also did not expect sensitivity to speaker informativeness to decrease across our age range. For variation between participants we used: $\sigma_i^\alpha \sim \text{Uniform}(0, 2)$.

For the global semantic knowledge parameters, we used $\mu_0^\theta \sim \text{Uniform}(-3, 3)$ for the intercept and $\mu_1^\theta \sim \text{Uniform}(0, 2)$ for the slope, because it is implausible to assume that semantic knowledge decreases with age. For the parameters capturing the variability the object specific trajectories around these overall parameters we used $\sigma_0^\theta, \sigma_1^\theta \sim \text{Uniform}(0, 2)$. Variation between participants: $\sigma_i^\theta \sim \text{Uniform}(0, 2)$.

To estimate the parameter distributions, we collected samples from six independent MCMC chains, collecting 750,000 samples from each chain and removing the first 250,000 for burn-in. Of the 500,00 remaining samples, we used every 10th to construct the posterior distribution. We excluded samples from two chains because they got stuck on a local maximum which resulted in parameter distributions that were substantially different from the other chains. The Appendix visualizes some examples of these distributions.

Model predicitons

We used the posterior distributions for the three parameters to generate a-priori model predictions for how the same participants should behave in the combination experiment. That is, we passed the parameter distributions estimated based on the four separate sensitivity experiments through our rational integration model to generate predictions for how children should behave in the combination experiment. Because our model parameters were specific to each child and familiar object, our model predictions were also specific to each trial in the combination experiment (i.e. unique combination of participant, familiar object and condition).

Remember that there were two conditions in the combination experiment, depending on how the mutual exclusivity and the common ground manipulations were aligned. In the *congruent* condition, the object in the prior social interaction (i.e., the discourse-familiar object) was the familiar object, and hence, both common ground (discourse novelty) and mutual exclusivity are cues to the same referent; in the *incongruent* condition, the object in the prior social interaction was the novel object, and hence, common ground (discourse novelty) and mutual exclusivity are cues to different referents.

Model evaluation

To evaluate the model predictions, we first replicated the group–level results of Bohn et al. (2021). The goal was to show that a) model predictions are highly correlated with the data and b) the *rational integration* model makes better predictions compared to a set of alternative models. Next, we turned to the focal participant–level analysis and evaluated how well the *rational integration* model predicted the trial-by-trail performance of each child, both on an absolute level and in comparison to the alternative models.

Alternative models

In addition to the *rational integration* model, we considered two alternative, lesioned models. Both models represent thy hypothesis that children focus on one type of inference (mutual exclusivity or common ground) whenever multiple inferences are possible. The predictions of these alternative models were generated using the same parameters as the *rational integration* model, the difference lies in which parameters were used.

The *no speaker informativeness* model assumed that the speaker does not communicate in an informative way. This corresponds to $\alpha_i = 0$, which causes the likelihood term to always be 1. As a consequence, this

model also ignores semantic knowledge (which affects the likelihood term) and the predictions of this model correspond to the prior distribution over objects:

$$P^{no\_si}_{L_1}(r \mid u; \{\rho_i\}) \propto P(r \mid \rho_i) \tag{4}$$

On the other hand, the *no common ground* model ignores common ground information, ρ_i. This model takes in object specific semantic knowledge and speaker informativeness but uses a prior distribution over objects that is constant across alignment conditions and uniform (e.g., [0.5, 0.5]). This model corresponds to a listener who only focuses on the mutual exclusivity inference and ignores the common ground manipulation. As a consequence, the listener does not differentiate between the two common ground alignment conditions.

$$P^{no\_cg}_{L_1}(r \mid u; \{\alpha_i \, \theta_{ij}\}) \propto P_{S_1}(u \mid r; \{\alpha_i, \theta_{ij}\}) \tag{5}$$

Correlation between model predictions and data

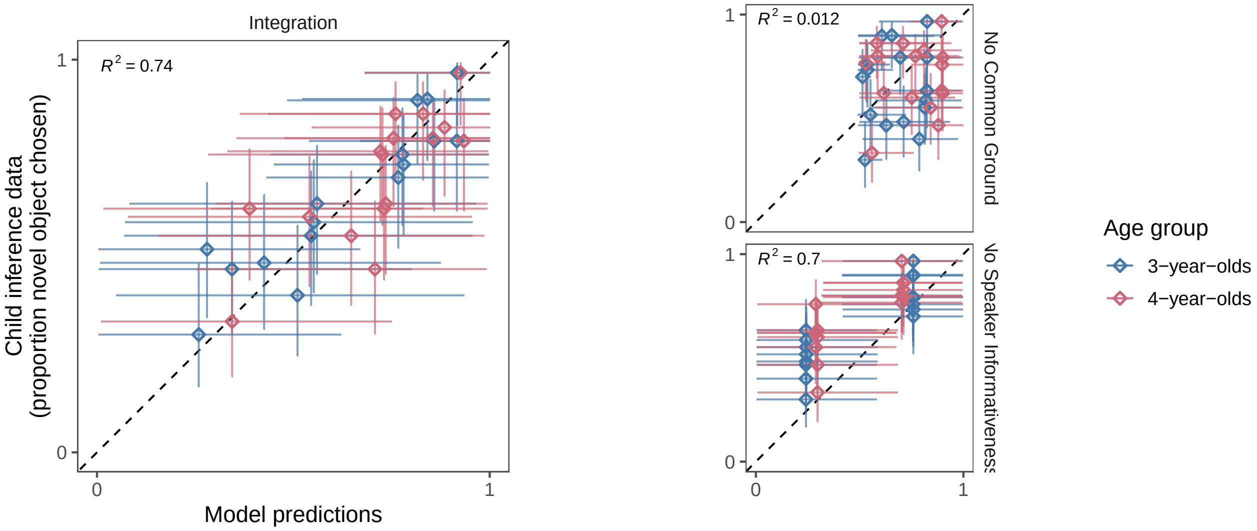

Figure 5: Correlation between model predictions and data (aggregated across individuals and binned by year with 95%HDI) for each trial in the combination experiment for the rational integration model (left) and the two alternative models (right).

As a first step, we correlated the model predictions with the data. To do so, we aggregated both the model predictions and the data for each trial (each with a different familiar object, half in the congruent and the other half in the incongruent condition) within each age group. Figure 5 shows a strong correlation between model predictions and the data with the model explaining 74% of the variance int he data. This correlation was comparable in size to that in Bohn et al. (2021). The correlation was also higher for the *rational integration* compared to the lesioned models.

Pairwise model comparisons

Furthermore, we compared the three models via the marginal likelihood of the data. Here we also saw that the *rational integration* model fit the data best (Figure 6). For pairwise comparisons, we used the marginal likelihoods to compute Bayes factors in favor of the *rational integration* model. There was overwhelming evidence that the *rational integration* model outperformed the *no common ground* (BF_{10} = 9.1e+53) and the *no speaker informativeness* model (BF_{10} = 1.2e+44).

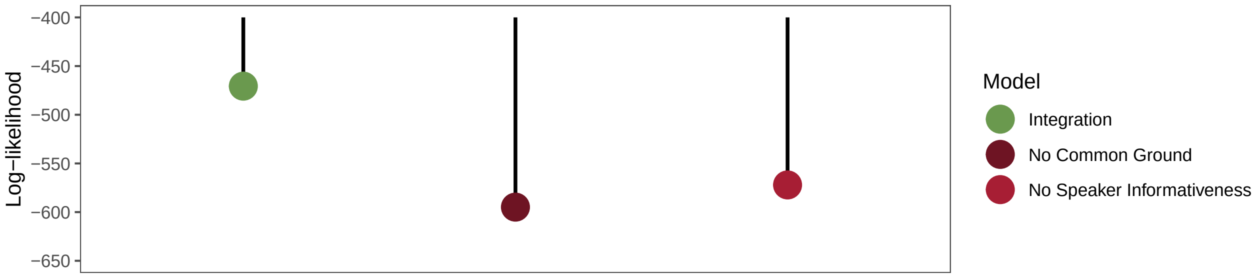

Figure 6: Log-likelihood of the data given each of the models in the model comparison.

Participant-specific model predictions

The results in the last section showed that the *rational integration* model made accurately predicted the average performance in each age group. However, the main focus of the present study was to see if this model also made accurate predictions on an individual level. That is, we wanted to see how well we could predict the behavior of individual children on individual trials.

The participant and familiar object specific parameters allowed us to generate predictions for every single trial for each participant. These predictions were generated via the parameters estimated based on the sensitivity experiments. For each sample of the posterior distribution of these parameters, we generated a prediction for the combination experiment. As a consequence, there was not a single prediction for each trial, but a distribution of predictions. The data we collected, however, was binary because for each trial we coded whether the child picked the unfamiliar object or not. Thus, to evaluate how well the model predicted the child's behavior on a given trial, we had to convert the distribution of continuous model predictions into a binary prediction. To do so, we first computed the Maximum-a-posteriori (MAP) of the predictive distribution for each child and trial and then flipped a coin with the MAP as its weight. Figure 7 gives a schematic overview of this process.

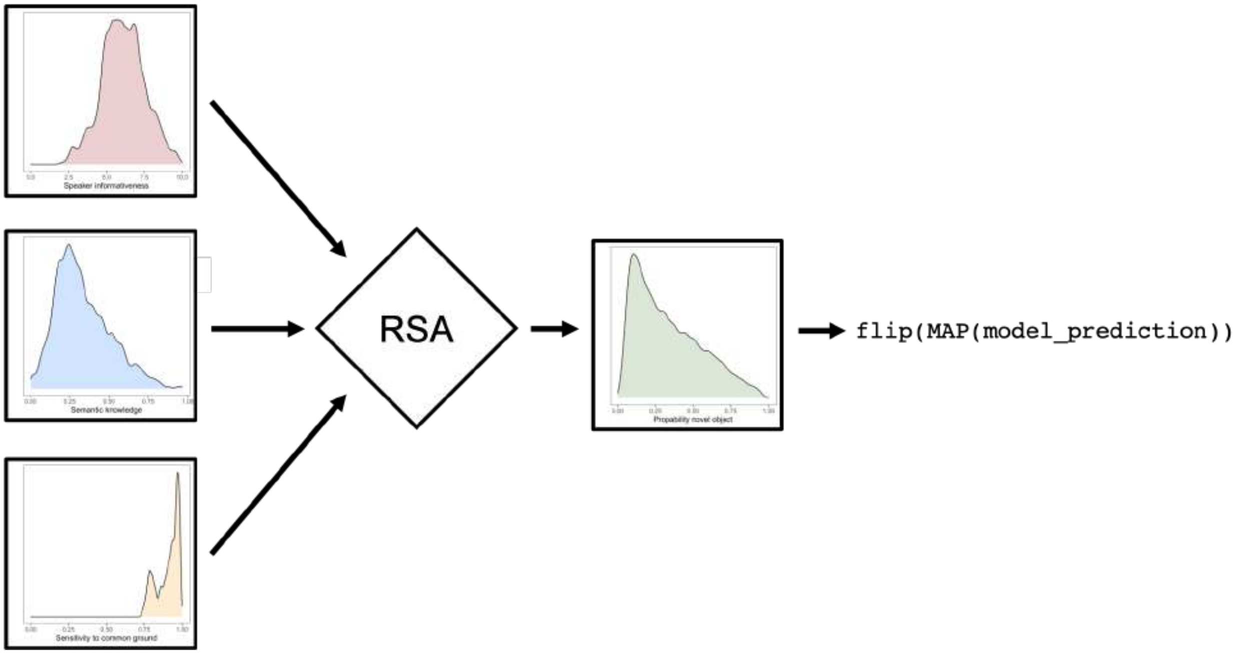

Figure 7: Schematic overview of how participant and trial specific model predictions were generated.

We then compared the model's prediction for a given trial to the data simply by asking whether the two

matched (coded as `1`) or not (coded as `0`). The number of matches (correct predictions) was then evaluated in three ways:

First, we averaged across predictions for each participant and compared the proportion of correct predictions across participants to a level expected by random guessing (50% correct predictions). Figure 8 shows the results of one run of the coin-flip procedure. For the analysis, we repeated the coin-flip 1000 times and for each run we computed the mean and the Bayes Factor for the comaprison to chance for each model. The *rational integration* model made correct predictions at a level clearly above chance for both age groups (correct onn average in 69% of cases, average Bayes Factor = 1.029054e+14). In a second step, we compared the predictions made by the *rational integration* model to those of the two lesioned models. For this, we generated trial and child specific model predictions for each of the lesioned models in the same ways as for the *rational integration* model. Here we saw that both models made predictions above chance (*no common ground* model: mean correct = 59%, mean BF = 208809; *no speaker informativeness* model: mean correct = 62%, mean BF = 367732.3), confirming that these were valuable alternative models. However, we also saw that the *rational integration* model was more likely to make correct predictions.

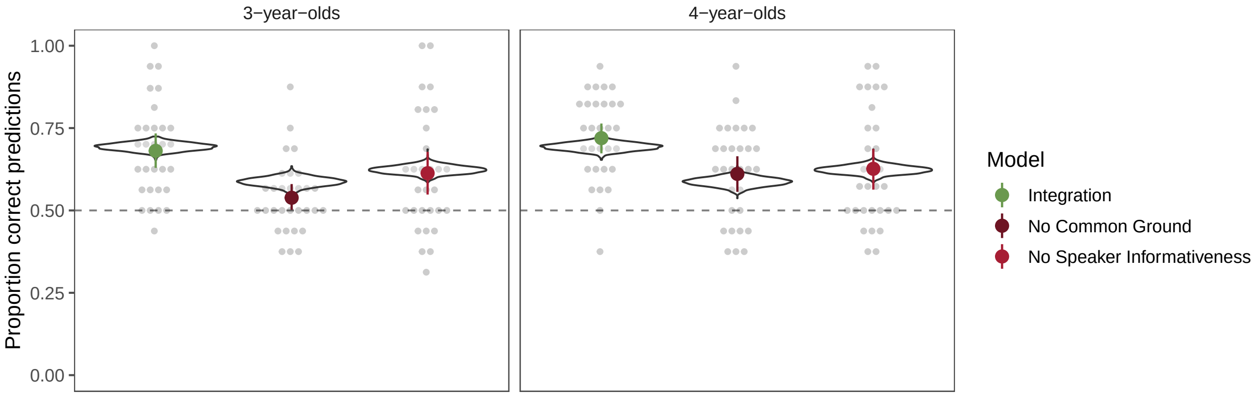

Figure 8: Proportion of correct model predictions for the three models (one iteration of coin-flip procedure). Violins show distribution of means for 1000 runs of the procedure. Colored dots show mean for each model (with 95% CI) and light grey dots show participant means.

Finally, we looked at the "hits" and "misses", that is, which responses the model was more likely to predict correctly and which not. Figure 9 showed that the *rational integration* model was – avergaed across 1000 runs of the coin-flip procedure – correct in almost all cases when the outcome was `1`. When the outcome was `0`, it was correct in about 50% of cases.

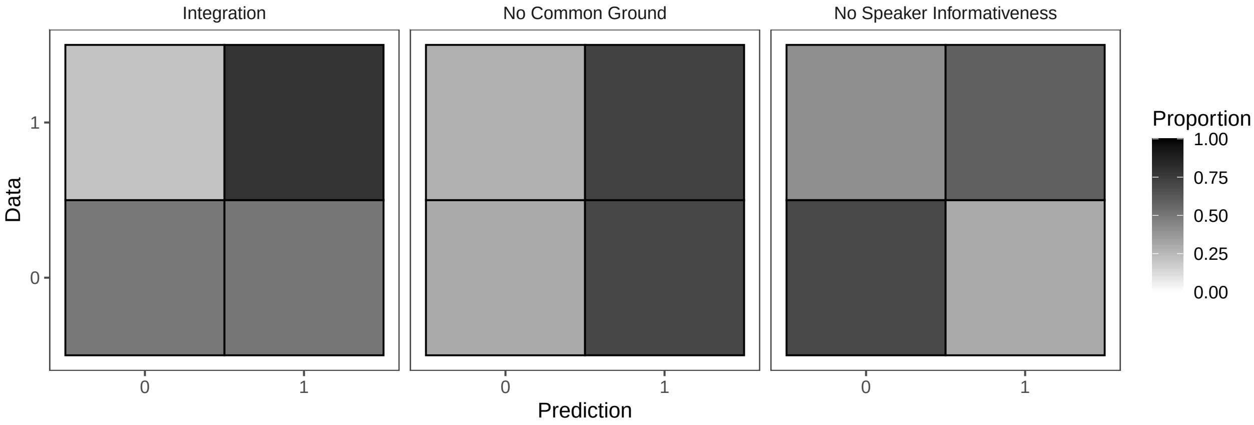

Figure 9: Proportion of correct predicitons by model for the two possible outcomes in the data.

Participant specific Bayes factors

For this final analysis, we repeated the model comparison on an individual level. That is, for each participant, we computed a Bayes factor (BF) in favor of the *rational integration* model compared to the two lesioned models. Figure 10A visualizes the distribution of the resulting BFs on the log-scale. Log-BFs larger than 0 correspond to BFs > 1 and thus suggest that the *rational integration* model fit the data better compared to the other model for this participant. Taken together, 0.6166667% of log-BFs were larger than 0 when comparing the *rational integration* model to the *no common ground* model and 0.6833333 % of log-BFs were larger than 0 when comparing the *rational integration* model to the *no speaker informativeness* model. Taken together, this suggests that for the majority of participants, the *rational integration* model was the best model to predict their behavior in a new situation. Figure 10B

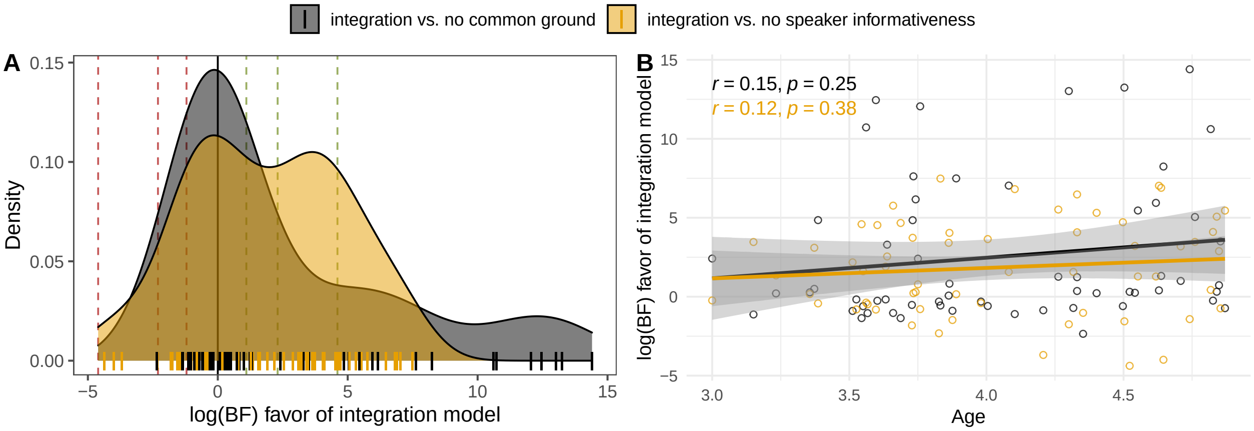

Figure 10: A: distribution of log Bayes Facotrs in favor of the integration model compared to the two lesion models. B: relation between age and log Bayes Factor in favor of the intergration model.

Appendix 1: Group model

In addition, we explored an alternative way of generating the participant specific predictions. Instead of using the participant specific parameters, we used the age specific hype parameters. In the section on model parameters we described how the participant specific parameters were estimated as deviations from age specific hyper parameters (i.e. values generated by an age sensitive slope and intercept). This alternative model uses parameters that were one level up in our hierarchical model. The reasoning was that these parameters might actually lead to better predictions, because they are estimated based on more data.

Figure 11 shows that this was not the case. The participant specific model was slightly more likely to make correct predictions compared to the group-level model.

Comparison ID vs. Group model

Appendix 2: Model parameters

Below we visualize the posterior distributions for some of the parameters in the model (speaker informativeness: Figure 12; semantic knowledge: Figure 13; sensitivity to common ground: Figure 14). Finally, we show the posterior distribution of the production probability parameter (Figure 15). This parameter was part of the production model and gives the probability that the child was able to produce a word if their semantic knowledge indicates that they knew it. This parameter was the same for all participants.

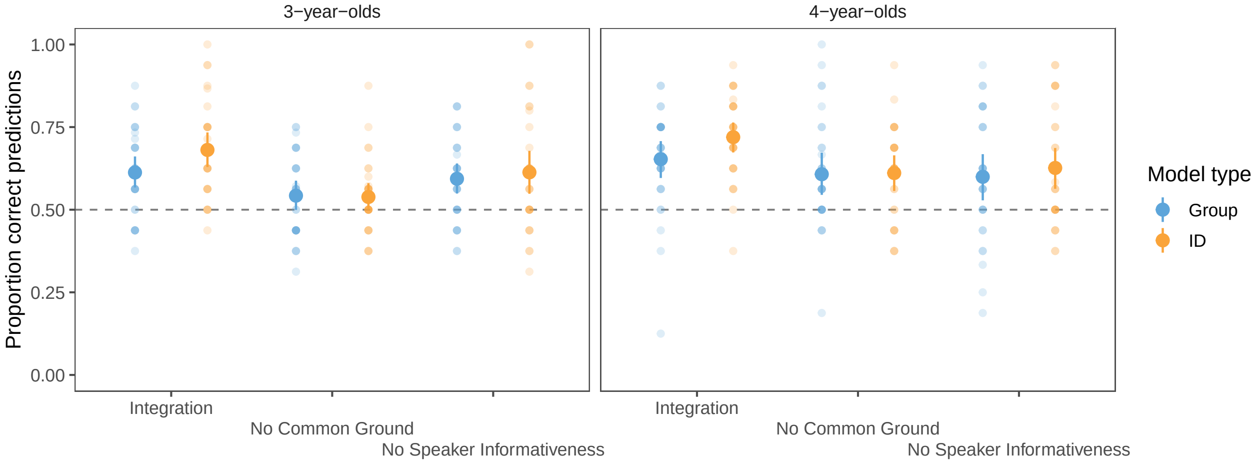

Figure 11: Proportion of correct model predictions for the two versions of the integration model. Group refers to predictions based on age specific hyper parameters and ID to predictions based on participant specific parameters. Colored dots show mean for each model (with 95% CI) and light colored dots show participant means.

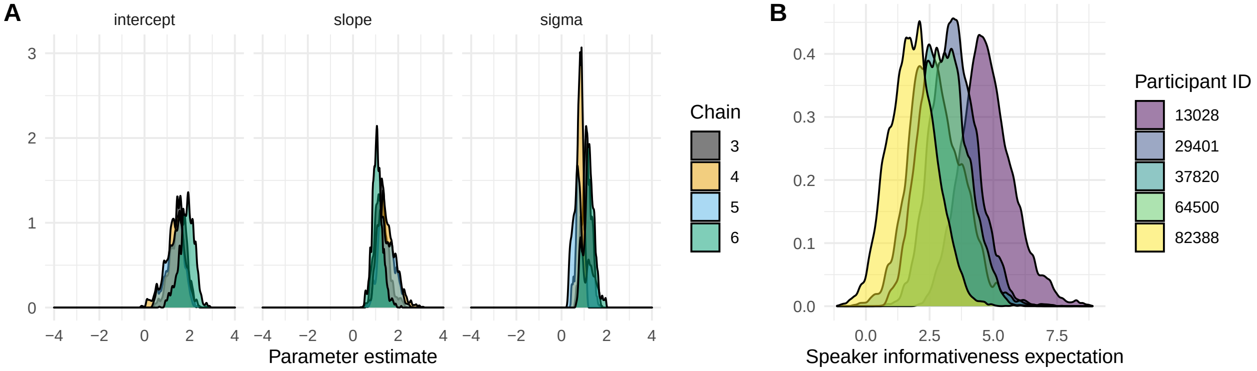

Figure 12: Model parameters for speaker informativeness. A) hyper parameters colored by MCMC chain and B) participant specific parameters for five randomly selected individuals.

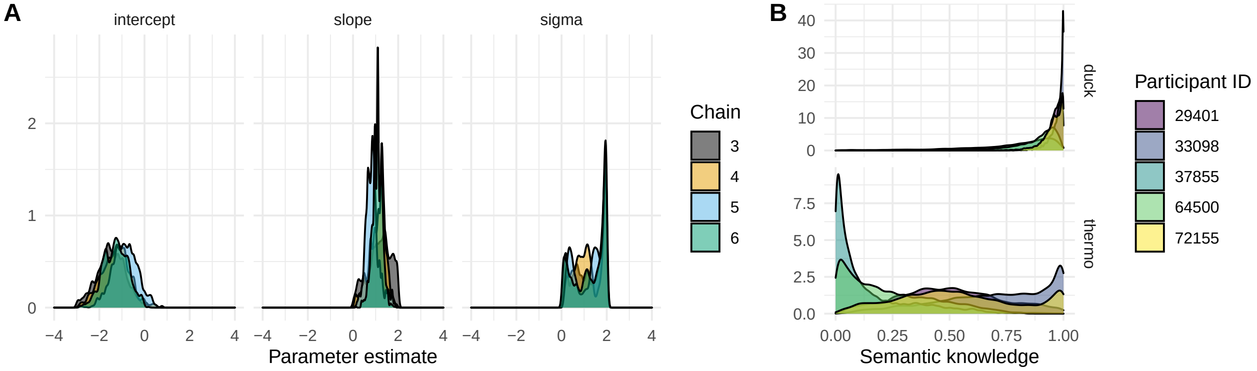

Figure 13: Model parameters for semantic knowledge. A) hyper parameters colored by MCMC chain and B) participant specific parameters for five randomly selected individuals for two familiar objects.

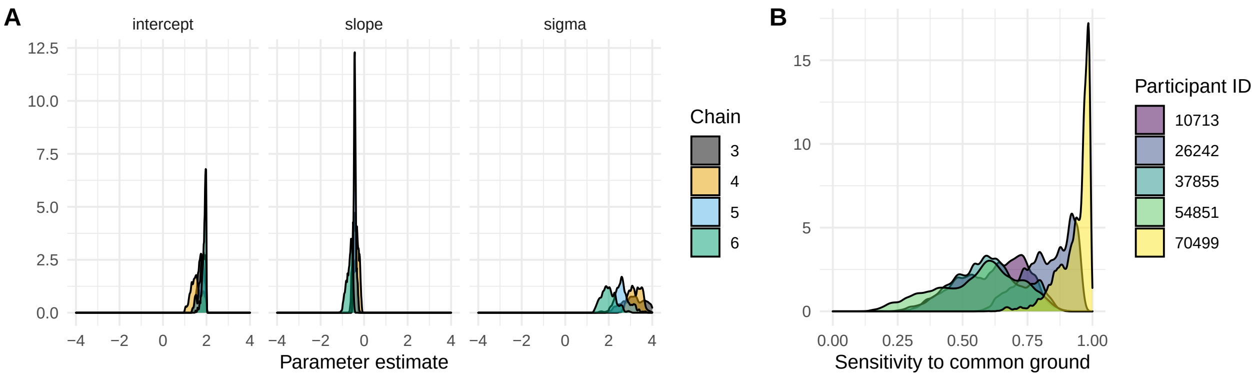

Figure 14: Model parameters for sensitivity to common ground. A) hyper parameters colored by MCMC chain and B) participant specific parameters for five randomly selected individuals.

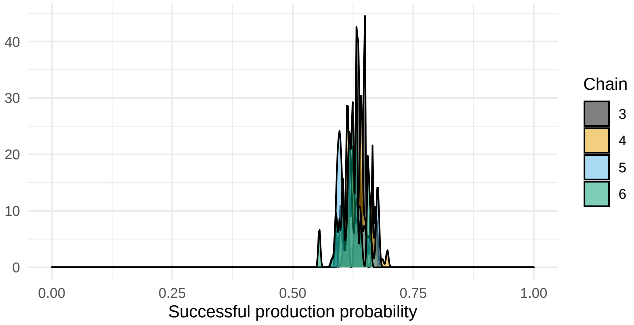

Figure 15: Model parameter for production probability colored by MCMC chain

References

Bohn, Manuel, Michael Henry Tessler, Megan Merrick, and Michael C Frank. 2021. "How Young Children Integrate Information Sources to Infer the Meaning of Words." *Nature Human Behaviour* 5 (8): 1046–54.

Frank, Michael C, and Noah D Goodman. 2012. "Predicting Pragmatic Reasoning in Language Games." *Science* 336 (6084): 998–98.

Goodman, Noah D, and Michael C Frank. 2016. "Pragmatic Language Interpretation as Probabilistic Inference." *Trends in Cognitive Sciences* 20 (11): 818–29.

Goodman, Noah D, and Andreas Stuhlmüller. 2014. "The design and implementation of probabilistic programming languages." http://dippl.org.

Grassmann, Susanne, Cornelia Schulze, and Michael Tomasello. 2015. "Children's Level of Word Knowledge Predicts Their Exclusion of Familiar Objects as Referents of Novel Words." *Frontiers in Psychology* 6: 1200.
